# Effect of circRNA_FOXO3 rs12196996 polymorphism and FOXO3 rs2232365 polymorphism on survival rate and severity of intensive care unit-acquired sepsis

**DOI:** 10.1080/21655979.2022.2034567

**Published:** 2022-02-13

**Authors:** Wang Lv, Zhang Wu, Yue Lin, Yingying Jiang, Xinguo Chen, Peng Zhu, Shengnan Wang

**Affiliations:** a Department of Emergency, Wenzhou People’s Hospital, Wenzhou, Zhejiang, China; b Department of Rheumatology and Immunology, Wenzhou People’s Hospital, Wenzhou, Zhejiang, China

**Keywords:** circRNA_FOXO3, rs12196996, FOXO3, rs2232365, survival, severity, ICU-acquired sepsis

## Abstract

The expression of circRNA_FOXO3 was found to be positively associated with the expression of Forkhead Box O3 (FOXO3), which is targeted and regulated by miR-23a. Polymorphisms in rs12196996 and rs2232365 have been reported in various diseases. In this study, we recruited intensive care unit (ICU)-acquired sepsis patients and grouped them according to their genotypes of rs12196996 and rs2232365. Quantitative real-time PCR was performed to analyze the expression of circRNA_FOXO3, FOXO3 mRNA, and miR-23a. ELISA was carried out to evaluate the abundance of cytokines and luciferase assay was used to explore the inhibitory role of miR-23a on circRNA_FOXO3 and FOXO3. Accordingly, we found that rs12196996 GG and rs2232365 AA were significantly correlated with prolonged survival of ICU-acquired sepsis patients. Rs12196996 GG and rs2232365 AA were also correlated with increased level of miR-23a, IL-10 and decreased level of TNF, IL-2, IFN, IL-6 and IL-1β in the peripheral blood cell samples of patients with ICU-acquired sepsis. The luciferase activity of wild-type (WT) circRNA_FOXO3 and FOXO3 were severely reduced by miR-23a. MiR-23a precursors could effectively suppress the expression of circRNA_FOXO3 and FOXO3 in the cells. Moreover, LPS-induced cell viability loss and dysregulation of cytokines were effectively restored by the knockdown of FOXO3 or circRNA_FOXO3 siRNA in the cells. This study revealed that the minor allele of rs12196996 polymorphism and rs2232365 polymorphism collaboratively contributed to the increased survival and suppressed severity of ICU-acquired sepsis.

## Graphical abstract

**Figure:**
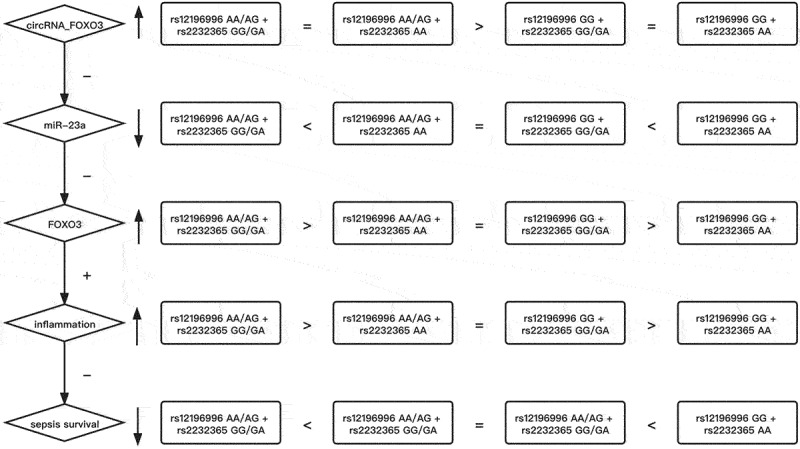

## Introduction

Severe sepsis and septic shock increase the frequency of admission into the hospital or make the medical treatment of seriously sick patients admitted in ICU more complicated, and the septic diseases are the most common causes of death of ICU patients currently [1–3]. Recent study points to the host immune pathways as the most important players in the pathophysiology of sepsis [1–4]. It is also involved in the progression of mild to severe condition, with severe condition defined as the loss of function of one organ, further spreading to multiple organs, and finally killing the patient due to septic shock. The complex communication, which involves both innate and adaptive immune response, resulted either in alleviation of sepsis or in several body organ dysfunction eventually leading to death [4].

Circular RNAs (circRNAs) are a type of transcripts that bind covalently and form a continuous loop [5]. Their expression can be correlated to a specific tissue location or to a progression stage of the disease [6]. CircRNAs act as miRNA sponges to regulate gene expression [7]. It has been shown that the circRNA_FOXO3 inhibited cell cycle progression as it makes a ternary complex with p21 and CDK2 [8].

FOXO3A like its counterparts FOXO1, FOXO6, FOXO4, and FOXO3 make FOXO family which functions as transcription factors. FOXO3A regulates cellular processes, such as autophagy, immune reaction, apoptosis as well as cell cycle arrest [9,10]. Correlation between stimulation of GSK‐3β and FOXO3A in regulation of growth of hepatoma cells was previously reported [11]. However, the nature of this correlation in endotoxin-induced cardiac muscle injury has not been reported previously. FOXP3 gene that contains both coding and non-coding exons is present on the p arm of the X chromosome [12]. Moreover, the gene was majorly found involved in regulation of immune cells like thrombocytes and peripheral T cells [13]. Previous studies have shown that the gene functioned as an important target for formation and replication of T-reg cells therefore, mutation in the gene could not only produce defective T-reg cells but also hinder transformation of nascent T-cells to T-reg cells [14]. FOXO3 gene was shown to encode both linear and circular version of the protein [15].

The circ_FOXO3 was related to the poor prognosis in acute myeloid leukemia (ALM) patients. This study found that CAD-associated polymorphism, SNP rs12196996, was present adjacent to the intron of circFOXO3 which could impact expression level of circFOXO3. Hence, the patients showing rs12196996 polymorphism in the gene might be more likely to get CAD. The results of the study were in line with the previously reported studies which showed that female patients with GG genotype produced fivefold higher levels of FOXP3 compared to the healthy counterparts. Therefore, the GG genotype in the female patients increased the risk of tuberculosis in these patients. Overall, this study showed that 924 > G polymorphism is responsible for increased expression level of FOXP3 and susceptibility to TB. The expression of circRNA_FOXO3 has been reported to be positively associated with the expression of FOXO3 [16]. FOXO3 has been reported to be targeted and regulated by miR-23a [17,18]. Moreover, circRNA_FOXO3 has been reported to induce proliferation, migration, and immune regulation [19]. And the two SNPs, rs12196996 polymorphism located in circRNA_FOXO3 and rs2232365 polymorphism located in FOXO3 mRNA, have been reported to be involved in the pathogenesis of various diseases such as coronary artery disease [20], late-onset preeclampsia [21], tuberculosis [22] and urothelial carcinoma [19]. In this study, we hypothesized that the SNPs located in circRNA_FOXO3 and FOXO3 mRNA is associated with the survival and severity of ICU-acquired infections. Accordingly, we recruited ICU-acquired sepsis patients and grouped them according to their genotypes of rs12196996 and rs2232365, in the aim to study the association between circRNA_FOXO3 rs12196996 polymorphism or FOXO3 rs2232365 polymorphism and the survival or severity of ICU-acquired infection.

## Materials and methods

### Human subjects and sample collection

For this study, a total of 310 patients with ICU acquired sepsis were recruited divided them into four groups according to the genotypes at circRNA_FOXO3 rs12196996 and FOXO3 rs2232365 polymorphisms: 1. rs12196996 AA/AG + rs2232365 GG/GA (N = 102), 2. rs12196996 AA/AG + rs2232365 AA (N = 88), 3. rs12196996 GG + rs2232365 GG/GA (N = 72), 4. rs12196996 GG + rs2232365 AA (N = 48). The unique characteristics including age, male sex proportion, site of infection and type of infection were compared using one-way ANOVA among the four groups.

To determine the genotypes at circRNA_FOXO3 rs12196996 and FOXO3 rs2232365, a total of 310 peripheral blood samples of patients with ICU acquired sepsis were collected. Then, the genomic DNA of the samples was extracted and amplified using PCR. About 2 μl of the PCR product, obtained from each sample, were added to 8 μl of a denaturing loading buffer, and PCR reaction was carried out following the below conditions: pre-denaturation at 98°C for 10 minutes, and 40 cycles of denaturation at 94°C for 30 seconds, annealing at 55°C for 30 seconds, and final extension at 72°C for 1 minute. Later, 10 μl of sample from each patient samples were resolved by using a 10% polyacrylamide gel to ensure the integrity of the PCR product. Finally, the PCR products were purified by using an Agarose Gel PCR Clean-up assay system (Thermo Fisher Scientific, MA) in accordance with the assay instructions provided in the assay protocol of the provider’s assay kit manual, and then subjected to Sanger direct sequencing (BGI, Beijing, China) to determine the genotypes of circRNA_FOXO3 rs12196996 and FOXO3 rs2232365 in each sample. Institutional ethical committee of Wenzhou People’s Hospital has approved the protocol of this study. All patients have signed informed consent before the initiation of this study.

### RNA isolation and real-time PCR

Extraction of RNA (total RNA) was performed on each sample by utilizing an RNAiso Plus RNA extraction reagent (Takara, Tokyo, Japan) following the specific manufacture’s protocol. Then, the isolated RNA from each sample was reverse transcribed into cDNA templates by making use of a iScript™ cDNA Synthesis Kit (Life Science Research, Hercules, CA) along with a Reverse Transcription kit (Thermo Fisher Scientific, MA) following assay protocols provided by manufacturers along with the assay kit. Then, qRT-PCR was performed using a Fast Start Universal SYBR Green Master Mix assay kit (Roche, Basel, Switzerland) in accordance with the specific assay protocol provided by the assay kit manufacturer. The real-time PCR reaction was done on an ABI Prism 7900HT real-time PCR machine (Applied Biosystems, Foster City, CA) using U6 as well as GAPDH as the internal control for the normalization of measured relative expression of target genes, i.e., circRNA_FOXO3 (Forward: 5’-GTGGGGAACTTCACTGGTGCTAAG-3’; Reverse: 5’-GGGTTGATGATCCACCAAGAGCTCTT-3), miR-23a (Forward: 5’-TTCCTGGGGATGGGATT-3’; Reverse: 5’-GAACATGTCTGCGTATCTC-3’), IL-6 (Forward: 5’-AGACAGCCACTCACCTCTTCAG-3’; Reverse: 5’-TTCTGCCAGTGCCTCTTTGCTG-3’), IL-1β (Forward: 5’-CCACAGACCTTCCAGGAGAATG-3’; Reverse: 5’-GTGCAGTTCAGTGATCGTACAGG-3’), and TNF-α (Forward: 5’-CTCTTCTGCCTGCTGCACTTTG-3’; Reverse: 5’-ATGGGCTACAGGCTTGTCACTC-3’).

### Cell culture and transfection

Human umbilical vein endothelial cells (HUVECs) were bought from the Shanghai Cell Bank at the Chinese Academy of Sciences (Shanghai, China). The cells were cultured in a RPMI 1640 medium added with 10% of fetal bovine serum (Gibco, Thermo Fisher Scientific, Waltham, MA) and suitable antibiotics. The culture conditions were saturated humidity, 37°C with 5% carbon dioxide. To further explore the regulatory network of circRNA_FOXO3 rs12196996 and FOXO3 rs2232365 polymorphisms, miR-23a and FOXO3, we cultured Human umbilical vein endothelial cells carrying the specific genotypes, and then transfected them with FOXO3 siRNA and miR-23a precursors, respectively. In brief, the Human umbilical vein endothelial cells carrying the specific genotype of the rs12196996 SNP were separated into three groups, i.e. 1. Negative control group (Human umbilical vein endothelial cells treated with 50 nM of a negative control siRNA); 2. FOXO3 siRNA group (Human umbilical vein endothelial cells treated with 50 nM of FOXO3 siRNA); and 3. miR-23a precursor group (Human umbilical vein endothelial cells treated with 50 nM of miR-23a precursors). Similarly, the Human umbilical vein endothelial cells carrying the AA genotype of the rs2232365 SNP were separated into four groups, i.e. a Negative control group (Human umbilical vein endothelial cells were treated with Negative control siRNA), a LPS plus Negative Control siRNA group (Human umbilical vein endothelial cells were treated with LPS followed by transfection of Negative Control siRNA), a LPS plus FOXO3 siRNA group group (Human umbilical vein endothelial cells were treated with LPS followed by transfection of FOXO3 siRNA), and a LPS+circRNA_FOXO3 siRNA group (Human umbilical vein endothelial cells were treated with LPS followed by transfection of circRNA_FOXO3 siRNA) The cells were transfected with corresponding siRNA/miRNA by using Lipofectamine 3000 (Thermo Fisher Scientific, MA) in accordance with the specific protocol include along with the transfection material. At 48 hours post procedure, analysis of target gene expression was carried out on the cells following harvesting.

Plasmids carrying miR-23a and FOXO3 were generated by GeneArt (Thermo Fisher Scientific, MA). The authors made sure that the density of cell was at 5 × 10^6^ cell/well. Later the cells were cultured for 12 h, pre-incubation of plasmids was carried out using After the cells were cultured overnight, the plasmid were pre-incubated with TransIT-LT1 (MirusBio, WI) for 15 min.

### Luciferase assay

Our sequence analysis indicated that miR-23a has a binding site on the 3’ UTR of FOXO3. In order to examine whether circRNA_FOXO3 rs12196996 and FOXO3 rs2232365 polymorphisms affect the expression of FOXO3, the 3’ UTR of FOXO3 carrying the A and G allele of the circRNA_FOXO3 rs12196996 and FOXO3 rs2232365 polymorphisms were cloned to get FOXO3-G and FOXO3-A vectors, which were then transfected into Human umbilical vein endothelial cells along with miR-23a mimics by using Lipofectamine 3000 in using with the procedure obtained from the manufacturer of the transfection material. About 48 hours after the transfection procedure, the luciferase activity was analyzed using a Dual Luciferase reporter gene assay kit (Thermo Fisher Scientific, MA).

### ELISA

In order to detect the release of IL-10, TNF, IL-6, IL-2, IFN, IL-1β, and TNF-α, mouse IL-10 Quantikine Enzyme-linked immunosorbent assay (ELISA) Kit (R&D, MTA00B, MN, USA), mouse TNF Quantikine Enzyme-linked immunosorbent assay (ELISA) Kit (R&D, MLB00C), mouse IL-6 Quantikine Enzyme-linked immunosorbent assay kit, mouse IL-2 Quantikine Enzyme-linked immunosorbent assay kit, mouse IFN Quantikine Enzyme-linked immunosorbent assay kit, mouse IL-1β Quantikine Enzyme-linked immunosorbent assay kit, and mouse TNF-α Quantikine Enzyme-linked immunosorbent assay (ELISA) Kit (R&D, M6000B) were used. Human umbilical vein endothelial cells were plated in 96-well plate at a cell density of 3 × 10^3^ cells/well. The cells were cultured for 12 hours and then treated with LPS and other compounds and then top layer of the cell culture was collected and analyzed following the Enzyme-linked immunosorbent assay kit user manual. Also, after the above procedure the serum of the samples was collected and centrifuged from respective groups and the top layer was again analyzed for the IL-10, TNF, IL-6, IL-2, IFN, IL-1β, and TNF-α secretion.

### Statistical analysis

All outcomes were revealed as mean ± SD deviations. The statistical relevance of inter-group contrasts was performed by combined Student’s t-tests, and the statistical relevance between multiple groups was performed by one-way ANOVA followed by Tukey’s test as the post hoc test. The statistical analyses were performed making use of Prism 7.0 software (GraphPad, La Jolla, CA). P = < 0.05 showed statistical relevance.

## Results

### Differential expression of circRNA_FOXO3, miR-23a, and FOXO3 mRNA in the peripheral blood of patients with ICU-acquired sepsis

**Figure 1.**
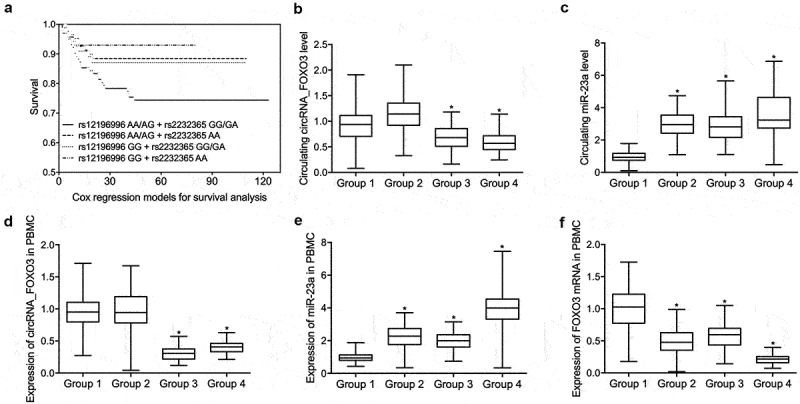
Differential expression of circRNA_FOXO3, miR-23a and FOXO3 mRNA in the peripheral blood of patients with ICU-acquired sepsis (one-way ANOVA and Tukey’s test, * P value < 0.05 vs. Group 1).

In this study, we hypothesized that the SNPs located in circRNA_FOXO3 and FOXO3 mRNA is associated with the survival and severity of ICU-acquired infections. Accordingly, we recruited ICU-acquired sepsis patients and grouped them according to their genotypes of rs12196996 and rs2232365, in the aim to study the association between circRNA_FOXO3 rs12196996 polymorphism or FOXO3 rs2232365 polymorphism and the survival or severity of ICU-acquired infection. The variables including age, male sex proportion, site of infection and type of infection of each participant were collected and compared among the four patient groups. As shown in Table 1, no remarkable difference was found. Peripheral blood samples were collected from patients with distinct genotypes at circRNA_FOXO3 rs12196996 and FOXO3 rs2232365. Cox regression models for survival analysis was performed to evaluate the survival of patients with distinct genotypes. The survival of patients with rs12196996 AA/AG + rs2232365 GG/GA was significantly decreased when compared with patients with rs12196996 AA/AG + rs2232365 AA and rs12196996 GG + rs2232365 GG/GA, while the survival of patients with rs12196996 GG + rs2232365 AA was notably increased when compared with patients with rs12196996 AA/AG + rs2232365 AA and rs12196996 GG + rs2232365 GG/GA (Figure 1a). RT-PCR was carried out to determine the level of expression of circRNA_FOXO3 and miR-23a in peripheral blood and peripheral monocytes (PBMCs). The expression of circRNA_FOXO3 was remarkably suppressed in the peripheral blood (Figure 1b) and PBMCs (Figure 1d) from patients with rs12196996 GG + rs2232365 GG/GA and rs12196996 GG + rs2232365 AA when compared with patients with rs12196996 AA/AG + rs2232365 GG/GA and rs12196996 AA/AG + rs2232365 AA. The level of miR-23a was significantly decreased in the blood samples and PBMCs from patients with rs12196996 AA/AG + rs2232365 GG/GA and increased in peripheral blood (Figure 1c) and PBMCs (Figure 1e) from patients with rs12196996 GG + rs2232365 AA when compared with patients with rs12196996 AA/AG + rs2232365 AA and rs12196996 GG + rs2232365 GG/GA. The expression of FOXO3 mRNA was highly increased in the PBMCs samples with rs12196996 AA/AG + rs2232365 GG/GA and decreased in the PBMCs (figure 1f) samples with rs12196996 GG + rs2232365 AA when compared with patients with rs12196996 AA/AG + rs2232365 AA and rs12196996 GG + rs2232365 GG/GA.

**Table 1.** Basic characteristics of recruited patients

Characteristics	rs12196996 AA/AG + rs2252365 GG/GA (N = 102)	rs12196996 AA/AG + rs2252365 AA (N = 88)	rs12196996 GG + rs2252365 GG/GA (N = 72)	rs12196996 GG + rs2252365 AA (N = 48)	P value
Age, years	60.3 ± 6.1	59.8 ± 4.7	58.2 ± 5.5	59.8 ± 6.4	0.615
Sex, male (%)	67 (65.7)	51 (58.0)	51 (70.8)	32 (66.7)	0.245
Site of infection (%)					0.470
Respiratory track	32 (31.4)	26 (29.5)	26 (36.1)	16 (33.3)	
Bloodstream	35 (34.3)	29 (33.0)	24 (33.3)	15 (31.3)	
Urinary track	5 (4.9)	5 (5.7)	3 (4.2)	2 (4.2)	
Skin/soft tissues	4 (3.9)	5 (5.7)	2 (2.8)	1 (2.1)	
Abdomen	1 (1.0)	1 (1.1)	2 (2.8)	1 (2.1)	
Type of infection (%)					0.504
Gram-positive infections	19 (18.6)	18 (20.5)	11 (15.3)	3 (6.2)	
Gram-negative infections	83 (81.4)	70 (79.5)	61 (84.7)	45 (93.8)

A: Cox regression models for survival analysis found that rs12196996 GG + rs2232365 AA was correlated with increased survival of patients with ICU-acquired sepsis than rs12196996 AA/AG + rs2232365 GG/GA.B: RT-PCR analysis found that rs12196996 GG was correlated with decreased expression of circRNA_FOXO3 in the peripheral blood of patients with ICU-acquired sepsis when compared with rs12196996 AA/AG.C: RT-PCR analysis found that rs12196996 GG + rs2232365 AA was correlated with increased expression of miR-23a in the peripheral blood of patients with ICU-acquired sepsis when compared with rs12196996 AA/AG + rs2232365 GG/GA.D: RT-PCR analysis found that rs12196996 GG was correlated with decreased expression of circRNA_FOXO3 in the PBMCs of patients with ICU-acquired sepsis when compared with rs12196996 AA/AG.E: RT-PCR analysis found that rs12196996 GG + rs2232365 AA was correlated with increased expression of miR-23a in the PBMCs of patients with ICU-acquired sepsis when compared with rs12196996 AA/AG + rs2232365 GG/GA.F: RT-PCR analysis found that rs12196996 GG was correlated with decreased expression of FOXO3 mRNA in the PBMCs of patients with ICU-acquired sepsis when compared with rs12196996 AA/AG.

### Differential expression of IL-10, TNF, IL-2, IFN, IL-6 and IL-1β in the peripheral blood of patients with ICU-acquired sepsis

**Figure 2.**
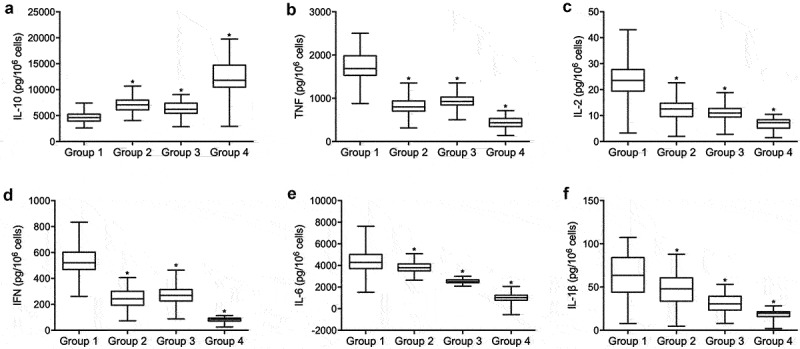
Differential expression of IL-10, TNF, IL-2 and IFN in the peripheral blood of patients with ICU-acquired sepsis (one-way ANOVA and Tukey’s test, * P value < 0.05 vs. Group 1).

The abundance of IL-10 was decreased in the PBMCs from patients with rs12196996 AA/AG + rs2232365 GG/GA and increased in the PBMCs from patients with rs12196996 GG + rs2232365 AA when compared with patients with rs12196996 AA/AG + rs2232365 AA and rs12196996 GG + rs2232365 GG/GA (Figure 2a). The abundance of TNF (Figure 2b), IL-2 (Figure 2c), IL-6 (Figure 2e), IL-1β (figure 2f) was significantly increased in the PBMCs from patients with rs12196996 AA/AG + rs2232365 GG/GA and decreased in the PBMCs samples with rs12196996 GG + rs2232365 AA when compared with patients with rs12196996 AA/AG + rs2232365 AA and rs12196996 GG + rs2232365 GG/GA. The abundance of IFN (Figure 2d) was significantly increased in the peripheral blood lymphocytes from patients with rs12196996 AA/AG + rs2232365 GG/GA and decreased in the peripheral blood lymphocytes from patients with rs12196996 GG + rs2232365 AA when compared with patients with rs12196996 AA/AG + rs2232365 AA and rs12196996 GG + rs2232365 GG/GA.

A: ELISA analysis found that rs12196996 GG + rs2232365 AA was correlated with increased expression of IL-10 in the PBMCs of patients with ICU-acquired sepsis when compared with rs12196996 AA/AG + rs2232365 GG/GA.B: ELISA analysis found that rs12196996 GG + rs2232365 AA was correlated with decreased expression of TNF in the PBMCs of patients with ICU-acquired sepsis when compared with rs12196996 AA/AG + rs2232365 GG/GA.C: ELISA analysis found that rs12196996 GG + rs2232365 AA was correlated with decreased expression of IL-2 in the PBMCs of patients with ICU-acquired sepsis when compared with rs12196996 AA/AG + rs2232365 GG/GA.D: ELISA analysis found that rs12196996 GG + rs2232365 AA was correlated with decreased expression of IFN in the peripheral blood lymphocytes of patients with ICU-acquired sepsis when compared with rs12196996 AA/AG + rs2232365 GG/GA.E: ELISA analysis found that rs12196996 GG + rs2232365 AA was correlated with decreased expression of IL-6 in the PBMCs of patients with ICU-acquired sepsis when compared with rs12196996 AA/AG + rs2232365 GG/GA.F: ELISA analysis found that rs12196996 GG + rs2232365 AA was correlated with decreased expression of IL-1β in the peripheral blood lymphocytes of patients with ICU-acquired sepsis when compared with rs12196996 AA/AG + rs2232365 GG/GA.

### miR-23a suppressed the expression of circRNA_FOXO3 and FOXO3

**Figure 3.**
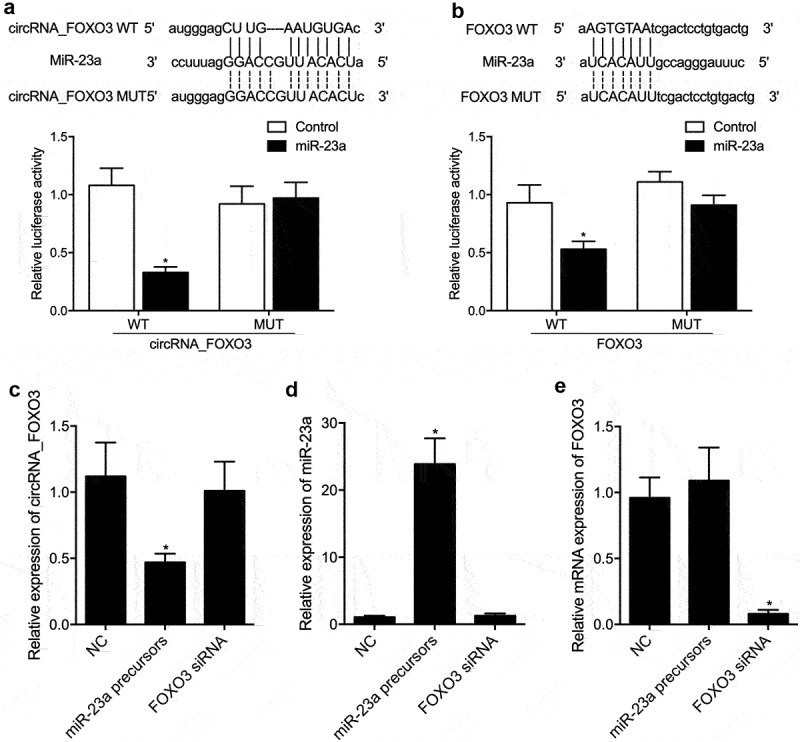
miR-23a suppressed the expression of circRNA_FOXO3 and FOXO3 (* P value < 0.05 vs. control group).

The luciferase activity of WT circRNA_FOXO3 was effectively suppressed by miR-23a (Figure 3a). The luciferase activity of wild-type FOXO3 was effectively suppressed by miR-23a (Figure 3b). Furthermore, we transfected miR-23a precursors and FOXO3 siRNA into HUVEC cells. The expression of circRNA_FOXO3 was repressed by miR-23a precursors (Figure 3c). The expression of miR-23a was effectively upregulated by miR-23a precursors in HUVEC cells (Figure 3d). The expression of FOXO3 mRNA was notably suppressed by FOXO3 siRNA in HUVEC cells (Figure 3e).

A: Sequence analysis and luciferase assay indicated that miR-23a repressed the luciferase activity of wild type circRNA_FOXO3 (one-way ANOVA and Tukey’s test).B: Sequence analysis and luciferase assay indicated that miR-23a repressed the luciferase activity of wild type FOXO3 (one-way ANOVA and Tukey’s test).C: RT-PCR analysis indicated that miR-23a precursors suppressed the expression of circRNA_FOXO3 in HUVECs cells (student’s t-test).D: RT-PCR analysis indicated that miR-23a precursors activated the expression of miR-23a in HUVECs cells (student’s t-test).E: RT-PCR analysis indicated that FOXO3 siRNA suppressed the expression of FOXO3 mRNA in HUVECs cells (student’s t-test).

### CircRNA_FOXO3 siRNA and FOXO3 siRNA restored the LPS induced dys-regulation of TNF-α, IL-1β, IL-6, miR-23a, circRNA_FOXO3 and FOXO3

**Figure 4.**
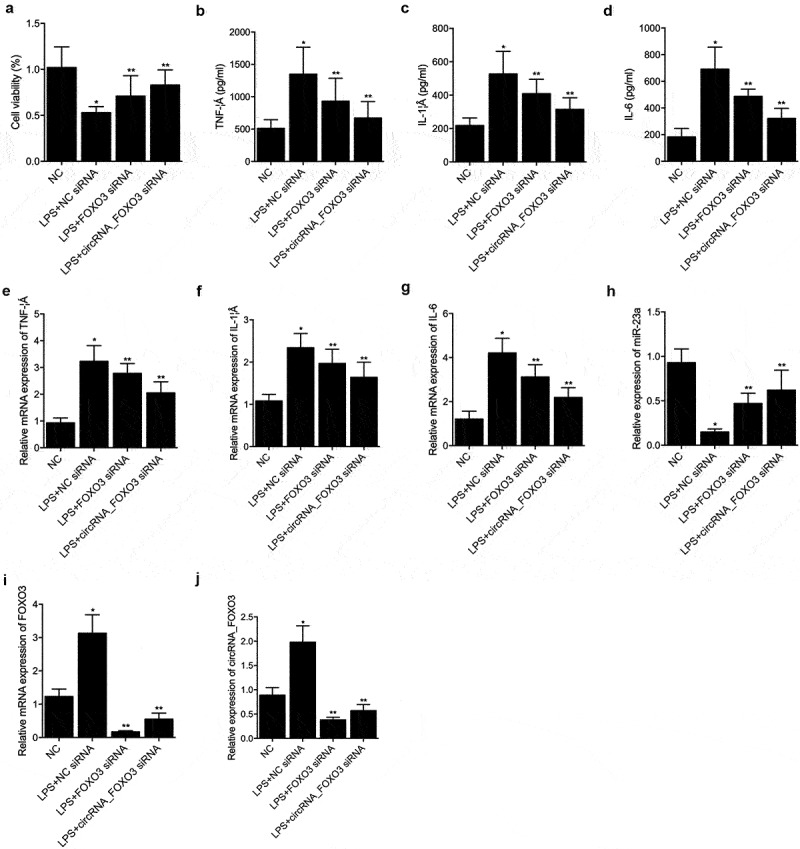
CircRNA_FOXO3 siRNA and FOXO3 siRNA restored the LPS induced dys-regulation of TNF-α, IL-1β, IL-6, miR-23a, circRNA_FOXO3 and FOXO3 (one-way ANOVA and Tukey’s test, * P value < 0.05 vs. NC group; ** P value < 0.05 vs. LPS+NC siRNA).

CircRNA_FOXO3 siRNA and FOXO3 siRNA showed considerable efficiency to restore the loss of cell viability after stimulation by LPS (Figure 4a). ELISA analysis indicated that LPS induced increase of TNF-α (Figure 4b), IL-1β (Figure 4c) and IL-6 (Figure 4d) was notably decreased by circRNA_FOXO3 siRNA and FOXO3 siRNA. RT-PCR analysis also showed that LPS induced increase of TNF-α (Figure 4e), IL-1β (figure 4f) and IL-6 (Figure 4g) was notably decreased by circRNA_FOXO3 siRNA and FOXO3 siRNA. LPS-induced decrease of miR-23a expression was apparently restored by circRNA_FOXO3 siRNA and FOXO3 siRNA (Figure 4h). It is worth noting that circRNA_FOXO3 siRNA showed a higher capability than FOXO3 siRNA in restoring the LPS-induced dysregulation of TNF-α, IL-1β, IL-6, and miR-23a. In addition, the LPS-induced up-regulation of FOXO3 mRNA (Figure 4i) and circRNA_FOXO3 (Figure 4j) was apparently decreased by circRNA_FOXO3 siRNA and FOXO3 siRNA to an extent that was even lower than in the control cells.

A: CCK-8 assay results indicated that the LPS induced cell viability loss of HUVECs cells was restored by circRNA_FOXO3 siRNA and FOXO3 siRNA.B: ELISA analysis indicated that the LPS induced TNF-α upregulation was restored by circRNA_FOXO3 siRNA and FOXO3 siRNA.C: ELISA analysis indicated that the LPS induced IL-1β upregulation was restored by circRNA_FOXO3 siRNA and FOXO3 siRNA.D: ELISA analysis indicated that the LPS induced IL-6 upregulation was restored by circRNA_FOXO3 siRNA and FOXO3 siRNA.E: RT-PCR analysis indicated that the LPS induced TNF-α mRNA upregulation was restored by circRNA_FOXO3 siRNA and FOXO3 siRNA.F: RT-PCR analysis indicated that the LPS induced IL-1β mRNA upregulation was restored by circRNA_FOXO3 siRNA and FOXO3 siRNA.G: RT-PCR analysis indicated that the LPS induced IL-6 mRNA upregulation was restored by circRNA_FOXO3 siRNA and FOXO3 siRNA.H: RT-PCR analysis indicated that the LPS induced decrease of miR-23a expression in HUVECs cells was restored by circRNA_FOXO3 siRNA and FOXO3 siRNA.I: RT-PCR analysis indicated that the LPS induced FOXO3 mRNA upregulation was restored by circRNA_FOXO3 siRNA and FOXO3 siRNA.J: RT-PCR analysis indicated that the LPS induced circRNA_FOXO3 upregulation was restored by circRNA_FOXO3 siRNA and FOXO3 siRNA.

## Discussion

ICU admitted patients who underwent a surgical treatment or needed a ventilating machine to support breathing were prone to hospital acquired infection [23,24]. Bacterial endotoxins or cytokines resulted in the septic shock [25,26]. Variation in the infection identifying system of patients partly explained differential response to similar infections, which could be due to single nucleotide polymorphisms (SNPs) in the system [27]. In this study, the patients with ICU-acquired sepsis were recruited and divided into four groups based on their genotypes at circRNA_FOXO3 rs12196996 and FOXO3 rs2232365. By performing Cox regression models for survival analysis, we found that GG genotype of rs12196996 GG and AA genotype of rs2232365 were correlated with enhanced survival of ICU-acquired sepsis patients.

Previous study had shown that the circRNA_FOXO3 was upregulated following the treatment with hydrogen peroxide, Cisplatin and Doxorubicin in cancer cells lines [28]. This study also showed that transfection of circRNA_FOXO3 siRNA decreased level of Foxo3 expressions [28]. The results of this study showed significant downregulation of Foxo3 in AML patients. Up-regulation of circRNA_FOXO3 stimulated apoptosis and reduced tumor development. Similar correlation was also observed in breast cancer cells [28]. The SNP rs12196996, which is present at the circRNA_FOXO3 adjacent to lateral intron could stimulate circular RNA expression compared to the linear RNA. This variation could impact a patient’s ability to contract CAD. In our study, we carried out qPCR to analyze the level of circRNA_FOXO3, miR-23a and FOXO3 mRNA in the peripheral blood of patients with ICU-acquired sepsis. Accordingly, we found that rs12196996 AA/AG was correlated with increased expression of circRNA_FOXO3 than rs12196996 GG. rs12196996 GG + rs2232365 AA was correlated with increased expression of miR-23a whereas rs12196996 AA/AG + rs2232365 GG/GA was correlated with suppressed expression of miR-23a. rs12196996 AA/AG was correlated with increased expression of FOXO3 mRNA than rs12196996 GG.

This study revealed that anti-inflammatory and antioxidative effects of nicotinamide were due to increased FoxO3 expression, which were in line with previously reported regulatory role for FoxO3 in the inflammation process both in cell studies and animal studies. In vivo studies in mice showed that FoxO3 knockdown increased cytokine production whereas increased level of FoxO3, specifically in T-cells, decreased cytokine production [29]. Infection caused due to bacteria blocked FoxO3 and over-stimulated cytokine production in the epithelial cells of intestine, whereas TNF-α-induced FoxO3 inactivation boosted IL-8 in HT-29 cells [30,31]. Moreover, we carried out luciferase assays to determine the effects of miR-23a on circRNA_FOXO3 and FOXO3 suppression. The expression of circRNA_FOXO3 and FOXO3 was significantly downregulated by miR-23a. FOXOs regulates number of genes and interacts with several different elements involved in cell fate process whereas GSK‐3β in turn stimulates expression of FoxO3A [30,32]. Even though the significance of FOXO3A in myocardial injury was unclear, FOXO3A had been shown to protect the cardiac function from hypertrophy pathology [33]. In contrast, the FOXO3A activated the pro-apoptotic genes (Bim, PUMA and Mxi1‐0), which induced cell apoptosis [34,35]. In order to explore the effects of knockdown of FOXO3A on expression of pro-inflammatory cytokines and cell apoptosis, siRNAs were used in this study. In this study, we found that the knockdown of FOXO3 or circRNA_FOXO3 effectively restored the dysregulation of TNF-α, IL-1β, IL-6, miR-23a, FOXO3 mRNA and circRNA_FOXO3 expression induced by LPS in HUVECs cells.

## Conclusion

In this study, we demonstrated that the minor allele of rs12196996 polymorphism located in circRNA_FOXO3 and the minor allele of rs2232365 polymorphism located in FOXO3 mRNA collaboratively contributed to the increased survival and suppressed severity of ICU-acquired sepsis.

## Data Availability

The data of this study are available from the corresponding author upon reasonable request.
